# IL-33 is negatively associated with the BMI and confers a protective lipid/metabolic profile in non-diabetic but not diabetic subjects

**DOI:** 10.1186/1471-2172-15-19

**Published:** 2014-05-10

**Authors:** Amal Hasan, Fahad Al-Ghimlas, Samia Warsame, Asma Al-Hubail, Rasheed Ahmad, Abdullah Bennakhi, Monira Al-Arouj, Kazem Behbehani, Mohammed Dehbi, Said Dermime

**Affiliations:** 1 Immunology and Innovative Cell Therapy Unit, Dasman Diabetes Institute, Kuwait City, Kuwait; 2 Fitness and Rehabilitation Centre, Dasman Diabetes Institute, Kuwait City, Kuwait; 3 Clinical Laboratory, Dasman Diabetes Institute, Kuwait City, Kuwait; 4 Biochemistry and Molecular Biology Unit, Dasman Diabetes Institute, Kuwait City, Kuwait; 5 Senior Management, Dasman Diabetes Institute, Kuwait City, Kuwait; 6 Biomedical Research Facility, King Khalid Medical City Center for Health Research, King Fahad Specialist Hospital Dammam, MBC-074, Bldg 100, Office 31, PO Box 15215, Dammam 31444, Kingdom of Saudi Arabia

**Keywords:** Interleukin-33, Atherosclerosis, HDL-C, LDL-C, TGL, BMI

## Abstract

**Objective:**

Recent studies have demonstrated a protective role for IL-33 against obesity-associated inflammation, atherosclerosis and metabolic abnormalities. IL-33 promotes the production of T helper type 2 (Th2) cytokines, polarizes macrophages towards a protective alternatively activated phenotype, reduces lipid storage and decreases the expression of genes associated with lipid metabolism and adipogenesis. Our objective was to determine the level of serum IL-33 in non-diabetic and diabetic subjects, and to correlate these levels with clinical (BMI and body weight) and metabolic (serum lipids and HbA1c) parameters.

**Methods:**

The level of IL-33 was measured in the serum of lean, overweight and obese non-diabetic and diabetic subjects, and then correlated with clinical and metabolic parameters.

**Results:**

Non-lean subjects had significantly (*P* = 0.01) lower levels of IL-33 compared to lean controls. IL-33 was negatively correlated with the BMI and body weight in lean and overweight, but not obese (non-diabetic and diabetic), subjects. IL-33 is associated with protective lipid profiles, and is negatively correlated with HbA1c, in non-diabetic (lean, overweight and obese) but not diabetic subjects.

**Conclusions:**

Our data support previous findings showing a protective role for IL-33 against adiposity and atherosclerosis, and further suggest that reduced levels of IL-33 may put certain individuals at increased risk of developing atherosclerosis and insulin resistance. Therefore, IL-33 may serve as a novel marker to predict those who may be at increased risk of developing atherosclerosis.

## Background

Obesity, characterized by an increase in adipose tissue mass, is a major risk factor for the development of a wide array of metabolic disorders including insulin resistance, glucose intolerance, and type 2 diabetes mellitus [1]. Obesity can be associated with increased concentrations of low density lipoprotein cholesterol (LDL-C) and triglycerides (TGL) [2], which play an important role in the development of atherosclerosis and coronary artery disease (CAD) [3-6]. Obesity is associated with a state of chronic low-grade inflammation, which plays a role in the development of both atherosclerosis [7] and diabetes [8]. In this regard, T cells and macrophages accumulate in the adipose tissue and secrete various proinflammatory cytokines and chemokines including tumour necrosis factor-alpha (TNF-α), interleukin-6, interleukin-8, C-reactive protein, plasminogen activator inhibitor-1, resistin, angiotensinogen and monocyte chemoattractant protein-1 [9,10]. Similarly, the accumulation of macrophage-derived foam cells [11,12] and T helper type 1 (Th1) cells within atherosclerotic plaques [13], and the resultant chronic inflammation of the arterial wall, plays a key role in plaque progression [14].

Interleukin-33 (IL-33), a newly identified cytokine of the interleukin-1 (IL-1) family, is broadly expressed in various tissue types [15,16] and is mainly present in stromal cells such as endothelial, epithelial and myocardial cells [16,17], as well as pre-adipocytes and adipocytes [18]. The receptor for IL-33 is ST2, which is a member of the IL-1 receptor family [15]. IL-33 has been shown to be proinflammatory in certain conditions such as allergy [19] and autoimmunity [20] and to be protective in others such as obesity [18], atherosclerosis [21] and cardiac fibrosis [22].

The metabolic effects of IL-33 in obesity have mostly been investigated in murine models [16,18,21]. In this regard, studies have shown that IL-33 exerts protective metabolic effects [16,18] through several mechanisms: (1) IL-33 decreases the expression of resistin (a mediator that is responsible for the development of insulin resistance and type 2 diabetes mellitus [18]), (2) leads to the accumulation of protective Th2 cells and their cytokines, and (3) leads to the polarization of resident macrophages towards a protective alternatively activated phenotype (CD206+ M2) [16,18]. These findings were further supported by *in vitro* studies showing that the treatment of murine adipocytes with IL-33 induces the production of protective Th2 cytokines (mainly IL-5 and IL-13), reduces lipid storage and decreases the expression of genes associated with lipid metabolism and adipogenesis [16,18]. Moreover, IL-33 administration to diabetic obese (*ob/ob*) mice results in reduced adiposity and fasting glucose, as well as improved glucose and insulin tolerance [16,18]; whereas feeding ST2^-/-^ mice a high fat diet leads to increased body weight, fat mass, impaired insulin secretion and glucose regulation [16,18,21].

Similarly, studies in murine (ApoE^-/-^) models have reported a protective role for IL-33, when administered systemically, against the development of atherosclerosis and cardiovascular disease [21]. The protective properties of IL-33 are exerted by the induction of a potent instigation from a pro-atherogenic Th1 to a protective Th2 phenotype [21], and by the expansion of suppressive CD4^+^ FOXP3^+^ ST2L^+^ regulatory T cells [23]. In this regard, IL-33 increases Th2 (IL-4, IL-5, and IL-13) but decreases Th1 (IFN-γ) cytokine production, and increases total serum immunoglobulin-A (IgA), IgE and IgG_1_ but decreases IgG_2a_, which collectively indicate a switch from a Th1 to a Th2 phenotype [21]. Moreover, IL-33 blocks the differentiation of macrophage-derived foam cells [11], which are responsible for the formation of atherosclerotic plaques [12]. Mice treated intraperitoneally with IL-33 exhibit smaller atherosclerotic lesions in the aortic sinus and have fewer macrophages and T-cells [21]; whereas those treated with sST2 (a decoy receptor that neutralizes IL-33) develop larger atherosclerotic plaques when compared to controls [21]. Anti-oxidized LDL-C antibodies are thought to be protective against the development of atherosclerosis by enhancing the clearance of oxLDL-C from the circulation [24]. Indeed, a recent study has proposed a role for IL-33 in the induction of anti-ox-LDL-C antibodies [21]. Furthermore, mice treated with IL-33 produce elevated levels of protective anti-ox-LDL-C antibodies via an effect on B1 cells, which produce high levels of IgM autoantibodies.

Collectively, the aforementioned studies suggest a protective role for IL-33 against the development of obesity-associated inflammation and atherosclerosis. However, little is known about the role of IL-33 in human obesity and its associated anomalies such as atherosclerosis. In addition, there are no correlation studies between IL-33 and important clinical parameters such as body mass index (BMI), serum lipids, and HbA1c. Therefore, we sought to investigate whether obesity is associated with lower circulating levels of IL-33, and whether IL-33 is associated with clinical parameters. Herein, we show that IL-33 is reduced in non-lean subjects, and that IL-33 is negatively correlated with the BMI and body weight in lean and overweight, but not obese (non-diabetic and diabetic), subjects. In addition, we show that IL-33 is associated with protective lipid profiles, and is negatively correlated with HbA1c, in non-diabetic (lean, overweight and obese) but not diabetic subjects.

## Methods

### Subjects and clinical parameters

The study was approved by the ethical committee board of the Dasman Diabetes Institute of Kuwait. Non-diabetic (glycated hemoglobin A1c, HbA1c < 6.5) and diabetic (physician-diagnosed, HbA1c > 6.5) adult subjects were recruited, and written informed consents were obtained. Measurements of height, weight, waist, and hip-width were obtained and subjects were classified according to their body mass indices (BMI, kg/m^2^) into lean (BMI < 25), overweight (25 ≤ BMI < 30) and obese (30 ≤ BMI < 40). The waist-to-hip ratios were calculated, and the whole-body composition including percentage of body fat (PBF), soft lean mass (SLM) and total body water (TBW) were measured by the use of IOI 353 Body Composition Analyzer (Jawon Medical).

### Routine laboratory measurements

Peripheral blood was obtained and the fasting glucose and lipid profiles (TGL, total cholesterol, LDL-C, high density lipoprotein cholesterol (HDL-C)) were analyzed using the Siemens Dimension RXL chemistry analyzer (Diamond Diagnostics, Holliston, MA). The level of HbA1c was determined using Variant™ (Bio-Rad, Hercules, CA). Normal values for TGL (<1.7 mmol/l), total cholesterol (<5.2 mmol/l), LDL-C (<3.3 mmol/l) and HDL-C (>1.03 mmol/l) were determined based on the ⁄American Heart Association’ guidelines. The characteristics of the subjects are shown in Table 1.

**Table 1:** Physical and clinical characteristics of the study populations

**Characteristics**	**LN**	**OW**	**OBS**	**Total**
(a)				
**Number of subjects**	10	8	13	31
**Age range (Years)**	32.4 ± 9.2	36.75 ± 7.12	43.8 ± 12.8	38.7 ± 11.35
**Total body weight**	58.5 ± 11.9	80.2 ± 11.45	*85.4	77 ± 19.5
**Body mass index**	*22.95	28.2 ± 1.67	33.4 ± 2.66	28.54 ± 5.3
**HbA1c (%)**	5.38 ± 0.32	5.47 ± 0.34	5.72 ± 0.32	5.55 ± 0.35
**Fasting blood glucose (mmol/l)**	4.8 ± 0.45	5.05 ± 0.38	5.15 ± 0.36	5.015 ± 0.41
**Total cholesterol (mmol/l)**	5.14 ± 1.3	5.2 ± 0.65	5.27 ± 1.1	5.2 ± 1.03
**HDL-C (mmol/l)**	1.35 ± 0.34	1.32 ± 0.34	1.15 ± 0.26	1.26 ± 0.315
**LDL-C (mmol/l)**	3.2 ± 0.97	3.4 ± 0.76	3.6 ± 1.0	3.43 ± 0.93
**TGL (mmol/l)**	*0.715	1.1 ± 0.53	1.1 ± 0.41	1.02 ± 0.46
**Characteristics**	**DM LN**	**DM OW**	**DM OBS**	**Total**
(b)				
**Number of subjects**	1	2	12	15
**Age range (Years)**	40	53 ± 14.14	50 ± 11.7	49.5 ± 11.4
**Total body weight**	88.5	81.4 ± 11.17	95.25 ± 12.1	92.96 ± 12.2
**Body mass index**	24.77	28.97 ± 0.66	34.35 ± 2.8	33 ± 3.9
**HbA1c (%)**	8.5	6.95 ± 0.49	8.88 ± 1.75	8.6 ± 1.7
**Fasting blood glucose (mmol/l)**	5.6	7.22 ± 1.3	10.40 ± 4.7	9.66 ± 4.5
**Total cholesterol (mmol/l)**	3.6	4.52 ± 0.54	5.01 ± 1.1	4.85 ± 1.1
**HDL-C (mmol/l)**	1.46	0.91 ± 0.09	*1.17	*1.13
**LDL-C (mmol/l)**	1.9	2.85 ± 0.64	2.85 ± 1.2	2.78 ± 1.1
**TGL (mmol/l)**	0.57	1.55 ± 0.05	1.81 ± 0.88	1.69 ± 0.85

(a) Data represent non-diabetic subjects with an HbA1c < 6.5. (b) Data represent diabetic subjects with an HbA1c > 6.5. Data shown represent standard deviation of the mean (*data that were not normally distributed are shown as medians).

### Quantification of serum IL-33 using enzyme-linked immunosorbent assay (ELISA)

A blood volume of 5 ml was collected in vacutainer blood collection tubes and allowed to clot at room temperature for 45 minutes. Serum was obtained by centrifugation at 1000 g for 10 minutes, and then stored at -80°C until use. The concentration of serum IL-33 was measured using the DuoSet ELISA kit (detection limit of the kit is 23.44 pg/ml), as per manufacturers’ instructions (R&D Systems), with minor modifications (neat serum samples were added to a 96-well plate coated with anti-IL-33 capture antibody and incubated overnight at 4°C). IL-33 values that fell below the detection limit of the kit (< 23.44 pg/ml) were excluded from the analysis.

### Statistical analysis

Statistical analysis was performed using the GraphPad Prism software (La Jolla, CA, USA). To test whether data come from a 'Gaussian Distribution', the 'D'Agostino-Pearson omnibus normality test was performed; since IL-33 data were not normally distributed, the non-parametric Mann-Whitney test was applied to compare between medians. For correlation analysis, the non-parametric Spearman r tests were applied. A *P*-value of less than 0.05 was considered significant.

## Results

The level of serum IL-33 was tested in a total number of 140 subjects; however, as many as 94 subjects (67%) had IL-33 levels below the detection limit of the kit (< 23.44 pg/ml), and therefore, due to specificity/sensitivity concerns were excluded from the analyses. Only those (46 subjects, 32%) with IL-33 levels above the detection limit of the kit were included in the data analysis.

### IL-33 is reduced in non-lean subjects

**Figure 1:**
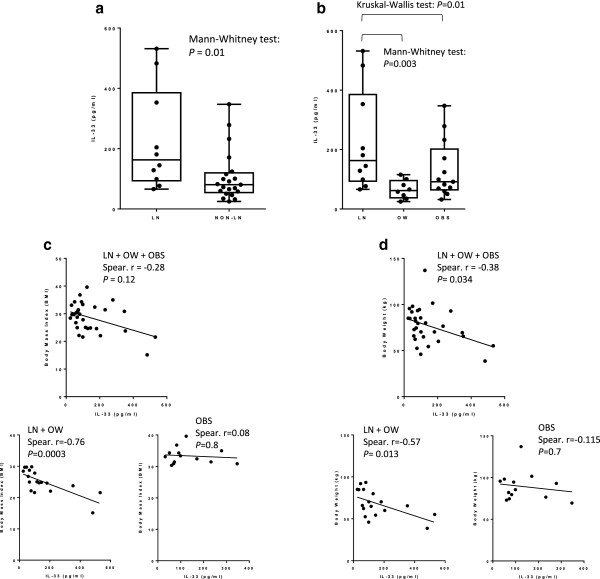
**IL-33 is reduced in non-lean subjects.** The concentration of serum IL-33 was measured in lean (n = 10) and non-lean (n = 21, 8 overweight and 13 obese) non-diabetic subjects. **(a)** Non-lean subjects had significantly (*P* = 0.01, non-parametric Mann-Whitney test) lower concentrations of IL-33 compared to lean controls. The graph shows the median of the 'minimum to maximum' IL-33 values. **(b)** Overweight subjects had significantly (*P* = 0.003, non-parametric Mann-Whitney test; the overall non-parametric Kruskal-Wallis test was *P* = 0.01) lower concentrations of IL-33 compared to lean controls. The graph shows the median of the ⁄minimum to maximum’ IL-33 values. **(c)** IL-33 is negatively correlated with the BMI in non-diabetic subjects, which was confirmed in lean/overweight (n = 18; Spearman r = -0.76, *P* = 0.0003) but not obese (n = 13; body weight: Spearman r = -0.115, *P* = 0.7; BMI: Spearman r = 0.08, *P* = 0.8) subjects. **(d)** IL-33 is negatively correlated with the body weight (n = 31; Spearman r = -0.38, *P* = 0.034) in non-diabetic subjects, which was confirmed in lean/overweight (n = 18; Spearman r = -0.57, *P* = 0.013) but not obese (n = 13) subjects.

The concentration of serum IL-33 was compared between lean (n = 10) and non-lean (n = 21, 8 overweight and 13 obese) non-diabetic subjects. As shown in Figure 1a, non-lean subjects had significantly (*P* = 0.01, non-parametric Mann-Whitney test) lower concentrations of IL-33 compared to lean controls; more specifically, the significant difference (*P* = 0.003, non-parametric Mann-Whitney test; the overall non-parametric Kruskal-Wallis test was *P* = 0.01) was obtained with overweight but not obese subjects (Figure 1b). Interestingly, diabetic obese subjects had higher levels of IL-33 compared to non-diabetic obese; however, it was not statistically significant (data not shown).

### IL-33 is negatively correlated with BMI and body weight in lean/overweight but not obese subjects

**Figure 2:**
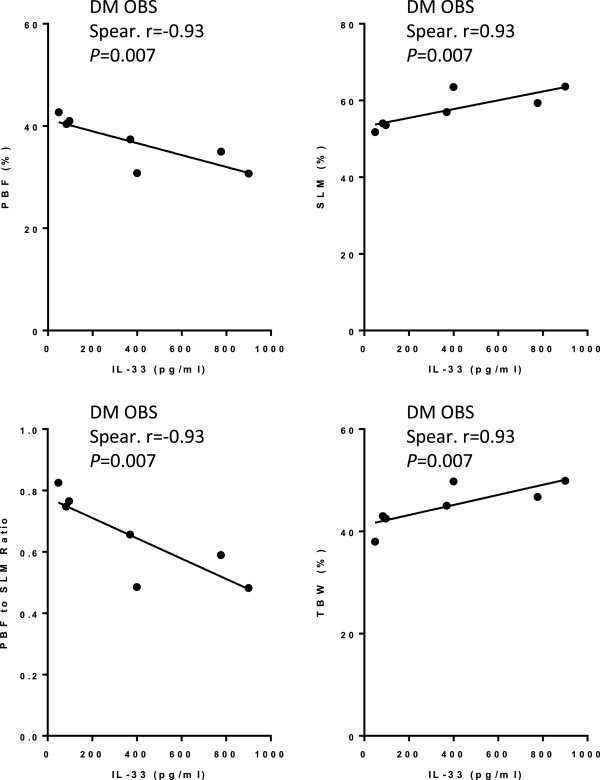
**IL-33 is negatively correlated with PBF and PBF to SLM ratio but positively correlated with SLM and TBW in diabetic subjects.** IL-33 is negatively correlated with PBF (n = 7; Spearman r = -0.93, *P* = 0.007) and PBF to SLM ratio (n = 7; Spearman r = -0.93, *P* = 0.007), but positively correlated with SLM (n = 7; Spearman r =0.93, *P* = 0.007) and TBW (n = 7; Spearman r =0.93, *P* = 0.007), in diabetic subjects.

IL-33 was negatively correlated with body weight (n = 31; Spearman r = -0.38, *P* = 0.034) in non-diabetic subjects. Importantly, the negative correlation was observed between IL-33 and body weight (n = 18; Spearman r = -0.57, *P* = 0.013) as well as BMI (n = 18; Spearman r = -0.76, *P* = 0.0003) in lean/overweight but not obese (n = 13; body weight: Spearman r = -0.115, *P* = 0.7; BMI: Spearman r = 0.08, *P* = 0.8) subjects (Figure 1c&d). Diabetic subjects were also analyzed for a possible correlation between IL-33 and BMI as well as body weight; however, since the diabetic group consisted mostly of obese subjects (obese n = 12, total n = 15), the analysis was confined to the obese group, which showed no correlation between IL-33 and BMI or body weight in diabetic obese (data not shown). Interestingly, in diabetic but not non-diabetic obese, IL-33 was found to be negatively correlated with PBF and PBF to SLM ratio (n = 7; Spearman r = -0.93, *P* = 0.007), and to be positively correlated with percentage of SLM and TBW (n = 7; Spearman r = 0.93, *P* = 0.007) (Figure 2). However, since the subject’s number was very low (n = 7), analysis of a larger group of subjects is required to verify this finding.

### IL-33 is associated with protective lipid profiles in non-diabetic but not diabetic subjects

**Figure 3:**
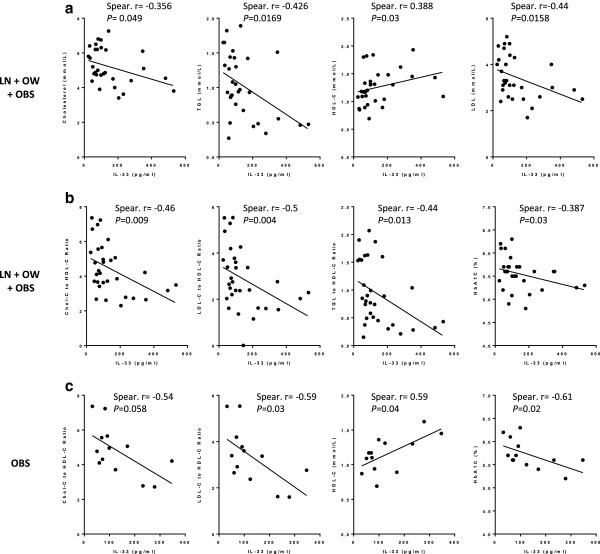
**IL-33 is associated with favorable serum lipid profiles and HbA1c in non-diabetic subjects. (a)** IL-33 is positively correlated with HDL-C (n = 31; Spearman r = 0.388, *P* = 0.03) but negatively correlated with total cholesterol (n = 31; Spearman r = -0.356, *P* = 0.049), LDL-C (n = 30; Spearman r = -0.44, *P* = 0.0158), and TGL (n = 31; Spearman r = -0.426, *P* = 0.0169) levels in non-diabetic subjects. **(b)** IL-33 is negatively correlated with cholesterol to HDL-C ratio (n = 31; Spearman r = -0.46, *P* = 0.009), LDL-C to HDL-C ratio (n = 31; Spearman r = -0.5, *P* = 0.004), TGL to HDL-C ratio (n = 31; Spearman r = -0.44, *P* = 0.013) and HbA1c (n = 31; Spearman r = -0.387, *P* = 0.03) in non-diabetic subjects. **(c)** IL-33 is negatively correlated with cholesterol to HDL-C ratio (n = 13; Spearman r = -0.54, *P* = 0.058), LDL-C to HDL-C ratio (n = 13; Spearman r = -0.59, *P* = 0.03) and HbA1c (n = 13; Spearman r = -0.61, *P* = 0.02), but positively correlated with HDL-C (n = 13; Spearman r = 0.59, *P* = 0.04), in non-diabetic obese subjects.

In non-diabetic subjects, IL-33 was positively correlated with HDL-C (n = 31; Spearman r = 0.388, *P* = 0.03) but negatively correlated with total cholesterol (n = 31; Spearman r = -0.356, *P* = 0.049), LDL-C (n = 30; Spearman r = -0.44, *P* = 0.0158), TGL (n = 31; Spearman r = -0.426, *P* = 0.0169) (Figure 3a), cholesterol to HDL-C ratio (n = 31; Spearman r = -0.46, *P* = 0.009), LDL-C to HDL-C ratio (n = 31; Spearman r = -0.5, *P* = 0.004) and TGL to HDL-C ratio (n = 31; Spearman r = -0.44, *P* = 0.013) (Figure 3b). When non-diabetic obese subjects were analyzed separately, an almost negative correlation was found between IL-33 and cholesterol to HDL-C ratio (n = 13, Spearman r = -0.54, *P* = 0.058) and LDL-C to HDL-C ratio (n = 13; Spearman r = -0.59, *P* = 0.03), and a positive correlation was found between IL-33 and HDL-C (n = 13; Spearman r = 0.59, *P* = 0.04) (Figure 3c).

**Figure 4:**
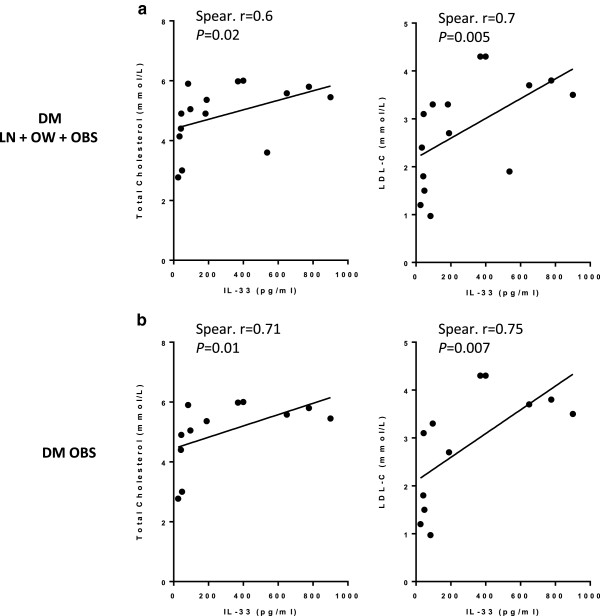
**IL-33 is positively correlated with total cholesterol and LDL-C in diabetic subjects. (a)** IL-33 is positively correlated with total cholesterol (n = 15; Spearman r = 0.6, P = 0.02) and LDL-C (n = 15; Spearman r = 0.7, P = 0.005) levels in diabetic subjects. **(b)** IL-33 is positively correlated with total cholesterol (n = 12; Spearman r =0.71, P = 0.01) and LDL-C (n = 12; Spearman r = 0.75, P = 0.007) levels in obese diabetic subjects.

In diabetic subjects, a positive correlation was found between IL-33 and total cholesterol (n = 15, Spearman r = 0.6, *P* = 0.02) as well as LDL-C (n = 15; Spearman r = 0.7, *P* = 0.005) levels (Figure 4a). Similarly, when diabetic obese subjects were analyzed separately, a positive correlation was found between IL-33 and total cholesterol (n = 12; Spearman r =0.71, *P* = 0.01) and LDL-C (n = 12; Spearman r = 0.75, *P* = 0.007) (Figure 4b).

### IL-33 is negatively correlated with HbA1c in non-diabetic but not diabetic subjects

Since the aforementioned data showed that IL-33 is associated with a protective lipid profile in non-diabetic but not diabetic subjects, we sought to investigate whether there was any correlation between IL-33 and HbA1c in non-diabetic and diabetic subjects. In this regard, a negative correlation (n = 31; Spearman r = -0.387, *P* = 0.03) was found between IL-33 and HbA1c in non-diabetic (Figure 3b) but not diabetic subjects (data not shown). When non-diabetic obese subjects were analyzed separately, it was found that IL-33 is negatively correlated with HbA1c (n = 13; Spearman r = -0.61, *P* = 0.02) (Figure 3c).

## Discussion

Previous murine studies have shown a protective role for IL-33 against adiposity, obesity-associated inflammation, insulin resistance and type 2 diabetes mellitus [16,18]. The mechanisms by which IL-33 exerts these protective effects are not fully understood, and little is known about the role of IL-33 in human obesity and its associated complications. In the present study, we explored the concentration of serum IL-33 in lean, overweight and obese subjects (non-diabetic and diabetic), and performed correlation analysis between IL-33 and key clinical and metabolic parameters. We show for the first time that IL-33 is reduced in non-lean subjects, and that this reduction is more pronounced in the overweight compared to the obese. One possible explanation for the reduced levels of IL-33 in overweight subjects is the presence of increased levels of the decoy receptor sST2. Interestingly, a recent study has shown that although the level of serum IL-33 in morbidly obese subjects is unaltered, the level of sST2 is increased [25].

We also show that IL-33 is negatively correlated with the BMI and body weight in non-diabetic subjects, which was observed in lean/overweight but not obese subjects. Similarly, no correlation was found between IL-33 and BMI or body weight in diabetic obese. Interestingly, however, in diabetic but not non-diabetic obese, a negative correlation was found between IL-33 and PBF and PBF to SLM ratio, and a positive correlation with percentage of SLM and TBW; however, since the subject’s number was very low, a larger group of subjects need to be analyzed to verify this finding. Nonetheless, previous studies have shown increased concentrations of sST2 in diabetic patients [26]; thus, the lack of correlation between IL-33 and body weight or BMI in our diabetic subjects may have been due to increased sST2 levels.

It is well known that obesity can be associated with the development of atherosclerosis and CAD [6]. Increased concentrations of LDL-C and TGL play a major role in CAD development [2]. Previous studies on murine models have shown that IL-33 reduces the development of atherosclerosis through the induction of anti-oxidized LDL-C antibodies [21]. Here, we show that IL-33 is positively correlated with HDL-C and negatively correlated with total cholesterol, LDL-C, TGL, cholesterol to HDL-C ratio, LDL-C to HDL-C ratio and TGL to HDL-C ratio in non-diabetic subjects. When we analyzed non-diabetic obese separately, we also found a positive correlation between IL-33 and HDL-C and a negative correlation between IL-33 and cholesterol to HDL-C ratio and LDL-C to HDL-C ratio. These findings suggest that IL-33 may play a protective role against the development of atherosclerosis by means of promoting a protective lipid profile in non-diabetic subjects, an effect that is less pronounced in the obese. In contrast to non-diabetic subjects, diabetic subjects lacked any protective correlation between IL-33 and serum lipids, and interestingly, a positive correlation was found between IL-33 and total cholesterol as well as LDL-C levels. Similarly, when diabetic obese subjects were analyzed separately, a positive correlation was found between IL-33 and total cholesterol and LDL-C. Therefore, while non-diabetic obese have a protective lipid profile (although less pronounced than the non-obese), diabetic obese have a pro-atherogenic lipid profile; this may provide an explanation as to why diabetic subjects are at increased risk of developing dyslipidemia and atherosclerosis. Indeed, insulin resistance is known to be an important factor in the development of atherosclerosis, and it has been reported that 80% of diabetic patients fail to achieve lipid-lowering goals [27].

Little is known about the molecular mechanisms that are involved in the development of insulin resistance [28]. Here, we show that IL-33 is negatively correlated with HbA1c in non-diabetic (including obese) but not diabetic subjects. This suggests that IL-33 may play a role in glucose regulation and thus may be involved in the molecular mechanisms that protect against the development of insulin resistance. This is in line with previous findings showing that the administration of IL-33 to diabetic obese mice reduces fasting glucose and improves glucose and insulin tolerance [16,18].

### Weakness of the data

A major limitation of the present study is the low number of subjects included in the data analysis due to the majority of subjects having IL-33 levels below the detection limit of the kit, which weakened the conclusions that could be drawn from the study.

## Conclusion

Our data suggest that IL-33 is reduced in non-lean subjects, and that IL-33 is negatively correlated with the BMI and body weight in lean/overweight but not obese subjects. Furthermore, we show that IL-33 is associated with protective lipid profiles, and is negatively correlated with HbA1c, in non-diabetic (lean, overweight and obese) but not diabetic subjects. These data suggest that reduced levels of IL-33 may put certain individuals at increased risk of developing insulin resistance and atherosclerosis, and may serve as a novel marker to predict those who may be at increased risk of developing atherosclerosis. However, owing to the small number of subjects studied, we cannot draw definitive conclusions, and further mechanistic studies are warranted.

## Competing interest

The authors declare that they have no competing interests.

## Authors’ contributions

AH conceived, designed, carried out the experiments, analyzed the data, and wrote the manuscript. FA recruited the study subjects, obtained clinical data of the subjects, and provided the body composition data. AA provided the biochemical data of the study subjects. SW coordinated between the clinic and the research laboratory and maintained the subjects' clinical data. AB and MA provided clinical advice. RA, MD and KB commented on the article. SD coordinated the Obesity Research Program, conceived, designed and supervised the study, and critically revised and edited the manuscript. All authors read and approved the final manuscript.
